# Poly-Phthalocyanine–Doped Graphene Oxide Nanosheet Conjugates for Electrocatalytic Oxidation of Drug Residues

**DOI:** 10.3389/fchem.2021.633547

**Published:** 2021-12-07

**Authors:** Prince Chundu, Edith Dube, Ngceboyakwethu P. Zinyama, Mambo Moyo, Munyaradzi Shumba

**Affiliations:** Department of Chemical Sciences, Midlands State University, Gweru, Zimbabwe

**Keywords:** spontaneous reaction, real biological samples, amidation, poly-phthalocyanines, simultaneous electrodetection

## Abstract

Donor and acceptor phthalocyanine molecules were copolymerized and linked to graphene oxide nanosheets through amidation to yield electrocatalytic platforms on glassy carbon electrodes. The platforms were characterized using transmission electron microscopy (TEM), scanning electron microscopy (SEM), Fourier-transform infrared (FTIR) spectroscopy, UV/Vis spectroscopy, cyclic voltammetry, and electrochemical impedance spectroscopy. The fabricated electrochemical catalytic surfaces were then evaluated toward electrocatalytic detection of ascorbic acid and tryptophan. These were characterized by a wide linear dynamic range and low limits of detection and quantification of 2.13 and 7.12 µM for ascorbic acid and 1.65 and 5.5 µM for tryptophan, respectively. The catalytic rate constant was 1.86 × 10^4^ and 1.51 × 10^4^ M^−1^s^−1^ for ascorbic acid and tryptophan, respectively. The Gibbs energy for catalytic reactions was −17.45 and −14.83 kJ mol^−1^ depicting a spontaneous reaction on the electrode surface. The sensor platform showed an impressive recovery when applied in real samples such as fresh cow milk, in the range 91.71–106.73% for both samples. The developed sensor therefore shows high potential for applicability for minute quantities of the analytes in real biological samples.

## Introduction

**SCHEME 1:**
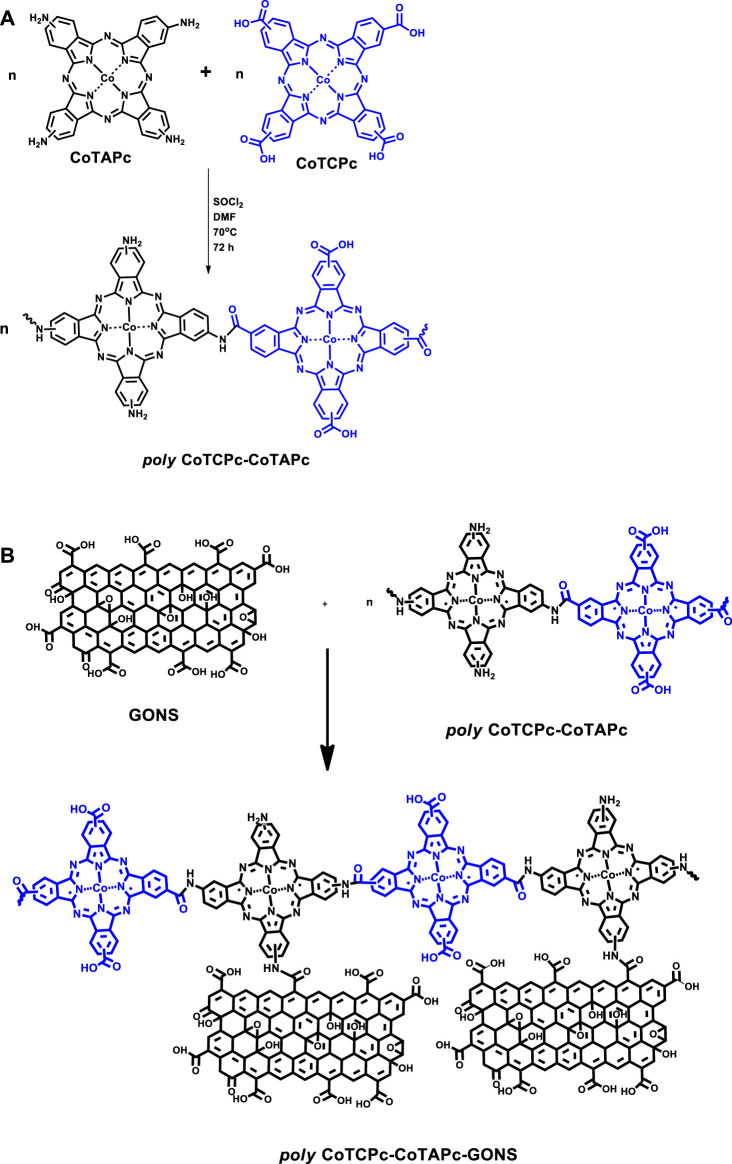
Prepolymerization of CoTAPc and CoTCPc **(A)**; conjugation of polymeric phthalocyanines to graphene oxide nanosheets **(B)**.

The electrocatalytic behavior of non-precious metals is enhanced when incorporated in macrocyclic ensembles such as phthalocyanines and porphyrins (Nyokong and Khene, 2016). These macrocyclics are very flexible to structure manipulation through different substituent introduction. The substituent can either pump or pull out electron density to or from the metal center. On the contrary, we have recently shown that the inclusion of carbon-based nanomaterials has resulted in improved electron flow and enhanced redox capabilities of the metal center in the macrocyclic ensemble (Shumba and Nyokong, 2016a). The approaches such as nanosizing and polymerization alike discourage aggregation which is prominent in phthalocyanines. Nanosizing encourages breaking down of the macrocyclics into smaller aggregates, while polymerization may not necessarily result in perfectly planar platforms, hence disrupting close proximity of subsequent layers in which cases aggregation is discouraged. The less aggregated the macrocyclics are, the more available the central metal is for electrocatalysis. Though polymerized phthalocyanines have been reported before (Nyokong and Khene, 2016; Liu et al., 2010; Sekhosana et al., 2017), this work explores for the first time prepolymerization of differently substituted phthalocyanines. This was achieved through amidation of carboxylate- and amine-terminated phthalocyanines as described before (Moyo et al., 2016; Shumba and Nyokong, 2017), Scheme 1A. Poly-phthalocyanines (Pcs) are thought to have a higher concentration of the electroactive metal center, hence an enhanced electrocatalytic performance compared to monophthalocyanines (MPcs) (Nyokong and Khene, 2016). The amine-terminated phthalocyanines and prepolymerized Pcs were further covalently linked to graphene oxide nanosheets before being used as electrode modifiers, Scheme 1B. It is noteworthy that the linear representation of the polymers in Scheme 1B is for convenience and does not rule out the most probable random coupling of the MPcs. Amidation linkage between metallophthalocyanines and carbon-based nanomaterials has recently been reported to form highly stable electrocatalysts compared to pristine phthalocyanines (Liu et al., 2013). Electropolymerized phthalocyanines and their subsequent application as electrocatalysts have been reported before (Griveau et al., 2002; Nyoni et al., 2015; Nemakal et al., 2019; Sajjan et al., 2019), and here, we report for the first time the prepolymerized phthalocyanines for the same purpose. The uniqueness in the present paper is that the monomeric forms are different unlike in previously reported electropolymerization steps where one monomeric form was utilized.

Electrochemical detection of different analytes is gaining popularity due to its fastness, simplicity, reproducibility, less simple preparation protocol, and inexpensive nature. The only challenge is the lack of meaningful sensitivity and high detection potentials characteristic of conventional electrodes. This has prompted the need to look for better electrocatalysts to improve the performance of conventional electrodes. One prerequisite of such modifiers should be their inexpensiveness and availability, hence the popularity gained by carbon-based nanomaterials.

We have reported the use of polymer-appended phthalocyanines (Mafuwe et al., 2018) and carbon nanotube–appended phthalocyanines (Shumba and Nyokong, 2017) among other manipulations, and we have shown how these can be used as electrode modifier materials. Normally, push–pull effects have been reported with respect to asymmetric phthalocyanines (Erdem et al., 2008; Dumrul and Yuksel, 2013), but we hereby demonstrate the same effects *via* copolymerized symmetric phthalocyanines with different sets of substituents. Amine- and carboxyl-terminated phthalocyanines are co-linked to form polymer phthalocyanines and/or linked to graphene oxide nanomaterials. In essence, we explore the effect of substituent groups on the electrocatalytic behavior of phthalocyanines.

Tryptophan and ascorbic acid, as small biomolecules, are used as test analytes because of their presence in most biological environments. Their simultaneous detection becomes critical since they coexist in physiological environments. The detection of these molecules is important for their biomedical chemistry, neurochemistry, and both diagnostic and pathological research (Kałużna-Czaplińska et al., 2019) since out-of-range concentrations are associated with some physiological disorders (Filik et al., 2016).

## Experimental

### Materials

Chemicals used in this study were of pure analytical grade and used directly without further purification unless stated. Potassium ferrocyanide {K_4_[Fe(CN)_6_]}, potassium ferricyanide {K_3_[Fe(CN)_6_]}, sodium hydroxide (NaOH), dimethylformamide (DMF), potassium chloride (KCl), hydrochloric acid (HCl), ascorbic acid (C_6_H_8_O_6_), l-tryptophan, cobalt chloride (CoCl_2_.6H_2_O), and potassium bromide (KBr) were obtained from ACE; methanol (CH_3_OH) and ethanol (C_2_H_5_OH) were purchased from Glassworld; and trimellitic acid anhydride (C_9_H_4_O_5_), ammonium chloride (NH_4_Cl), urea [CO(NH_2_)_2_], nitrobenzene (C_6_H_5_NO_2_), hydrochloric acid (HCl), thionyl chloride (SOCl_2_), tetrahydrofuran (THF), potassium dihydrogen phosphate (KH_2_PO_4_), ammonium molybdate [(NH_4_)_2_MoO_4_], sodium nitrate (NaNO_3_), potassium permanganate (KMnO_4_), hydrogen peroxide (H_2_O_2_), graphite flakes, sulfuric acid (H_2_SO_4_), and distilled water were locally prepared in the laboratory. The carboxylate-terminated (CoTCPc) and amine-terminated (CoTAPc) phthalocyanines (Moyo et al., 2016; Shumba and Nyokong, 2017) and graphene oxide nanosheets were synthesized as reported in the literature.

### Equipment

Transmission electron microscopy (TEM) images were obtained from a Zeiss Libra TEM 120 model operated at 90 kV. Scanning electron microscopy (SEM) images of modified glassy carbon plates (Goodfellow, UK) were obtained using a TESCAN Vega TS 5136LM electron microscope.

The ground-state electronic absorption was measured using a Shimadzu UV-2550 spectrophotometer.

Fourier-transform infrared (FTIR) spectroscopy (Nicolet 6700 model) was used in IR characterization.

All electrochemical work was done on an Autolab potentiostat PGSTAT 302N equipped with NOVA version 1.10 software and encompassed with three electrochemical cells comprising of a glassy carbon working electrode (GCE), platinum wire auxiliary electrode, and saturated Ag|AgCl reference electrode.

### Synthesis

Poly-CoTAPc-CoTCPc, poly-CoTAPc-CoTCPc-GONS, CoTAPc-GONS, CoTAPc, and GONS were covalently linked *via* an amide bond as reported before with minor modifications (Scheme 1) (Shumba and Nyokong, 2017). A mass of 0.02 g CoTAPc and 0.01 g GONS was added to 3 ml SOCl_2_ and 6 ml DMF, and the mixture was stirred at 70°C for 72 h. The resulting mixture was allowed to cool to room temperature and then centrifuged for 20 min at 4,000 rpm, and the supernatant was decanted. The CoTAPc-GONS solid was washed with tetrahydrofuran several times and then with ethanol to remove the tetrahydrofuran. The CoTAPc-GONS solid was dried at room temperature. Poly-CoTCPc-CoTAPc and poly-CoTCPc-CoTAPc-GONS were synthesized by adopting the just described procedure with equal amounts of each Pc and used as electrode modifiers. These were utilized to modify glassy carbon electrodes as described before, and the resultant electrodes were named GO/GCE, CoTAPc/GCE, CoTCPc/GCE, poly-CoTAPc-CoTCPc-GCE, poly-CoTAPc-CoTCPc-GONS/GCE, and CoTAPc-GONS/GCE.

## Results and Discussion

### Characterization

#### Spectroscopic Characterization

##### Fourier-Transform Infrared Spectra

**FIGURE 1:**
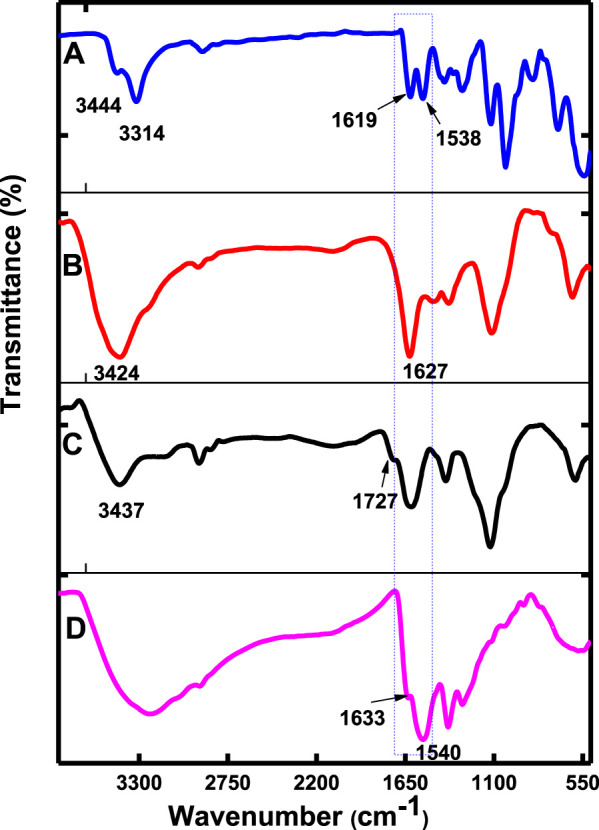
FT-IR spectra of CoTAPc **(A)**, CoTAPc-GONS **(B)**, GONS alone **(C)**, and poly-CoTCPc-CoTAPc-GONS **(D)**.

The FTIR spectra (Figure 1, using CoTAPc, CoTAPc-GONS, GONS, and poly-CoTCPc-CoTAPc-GONS, and Supplementary Figure S1, using CoTCPc and poly-CoTCPc-CoTAPc) were employed to prove amide bond formation between CoTAPc and GONS, CoTAPc and CoTCPc, and poly-CoTCPc-CoTAPc and GONS. The spectrum of CoTAPc alone (Figure 1A) displayed peaks at 3,444 and 3,314 cm^−1^ and split peaks at 1,619 and 1,588 cm^−1^, characteristic of primary amines. The spectrum of GONS (Figure 1C) displayed a peak at 3,437 cm^−1^ (OH) and shoulder peak at 1727 cm^−1^ (C=O) due to the presence of hydroxyl and carboxylic acid groups, respectively (Scheme 1B). Similar functional groups were displayed by CoTCPc when alone (Supplementary Figure S1A). The disappearance of the primary amine twin peaks and the appearance of the secondary carbon amide (O=C-NH) band which overlaps with the carbonyl group peak at around 1,627 cm^−1^ for CoTAPc-GONS (Figure 1B), and at 1,532 cm^−1^ for CoTCPc-CoTAPc (Supplementary Figure S1B), confirm the linkage of the carboxylic acid–functionalized complexes to amine-functionalized phthalocyanines through an amide bond. The spectra of poly-CoTCPc-CoTAPc-GONS (Figure 1D) show the secondary carbon amide peak at 1,540 cm^−1^ and hydroxyl and carboxyl groups at 3,300 and 1,633 cm^−1^, respectively.

##### Ground-State Absorption Spectra

**FIGURE 2:**
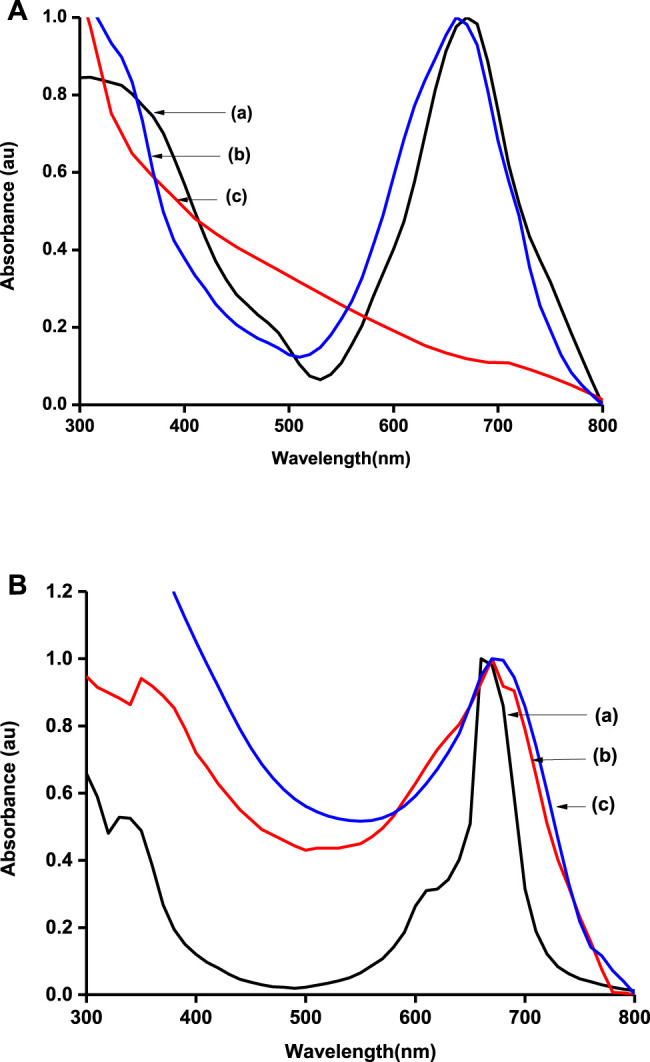
Normalized UV/Vis spectra of electrode modifiers: **(A)** CoTAPc (a), CoTAPc-GONS (b), and GONS (c); **(B)** CoTCPc (a), poly-CoTCPc-CoTAPc (b), and poly-CoTCPc-CoTAPc-GONS (c) in DMSO.

Ground-state absorption spectra were also run for the polymers and the carbonaceous materials (Figures 2A,B). The graphene materials produced a featureless spectrum that has been reported elsewhere (Ikram et al., 2020). MPcs were characterized by two main absorption regions, one around 300 nm (B-band) and the other above 600 nm (Q-band). The Q-band is attributed to the transition from the HOMO a_1u_ to the LUMO e_g_, while the B-band is attributed to the transition from the HOMO a_2u_ to the HOMO e_g_ in monomer units of the MPcs (Kaya et al., 2015). The B-band was however swamped in the presence of graphene sheets. Metallophthalocyanines are well known for their tendency to form aggregates in solution, and hence, a shoulder is observed alongside the Q-band (Choi et al., 2019; Ghazal et al., 2019). The Q-band for poly-CoTCPc-CoTAPc (Figure 2B(b)) is split probably because the polymer is made from two Pcs with different Q-band positions that slightly overlapped on polymerization.

### Microscopic Analyses

**FIGURE 3:**
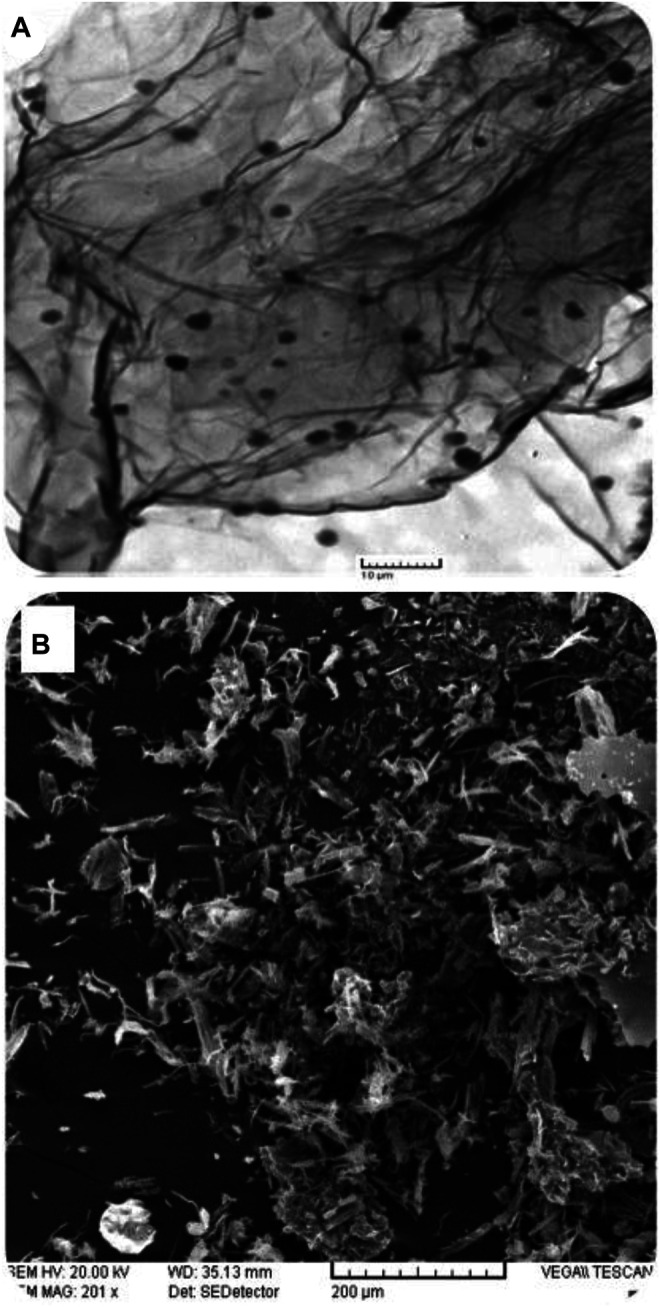
Microscopic characterization: **(A)** TEM micrograph of poly-CoTAPc-CoTCPc-GONS; **(B)** SEM image of poly-CoTAPc-CoTCPc-GONS/GCE.

The nature of electrode modification determines the surface area available for interface between the analyte and the redox platform. As such, electrode surface morphology plays a very vital role in electrochemical reactions. It is noteworthy though that morphology only becomes vital given the fabricated platform’s electron exchange capability. To this effect, scanning electron microscopy becomes a prerequisite to augment surface coverage determinations as a way to explore the nature of the sensing platform that ensues from such studies. The TEM micrograph shows the sheet structure of the graphene oxide nanosheets that are decorated by poly-phthalocyanines revealing as black spots spread over the sheet (Figure 3A). The SEM images show a rolled-up, flaked, and crumpled rough surface (Figure 3B). The rough structure of poly-CoTAPc-CoTCPc-GONS/GCE provides a high specific surface area and high electron transfer rate, confirmed by the higher electroactive electrode surface area as determined above.

### Electrochemical Characterization

**FIGURE 4:**
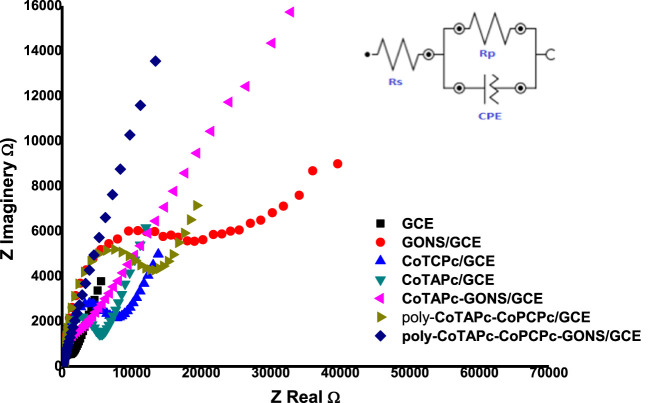
Nyquist plots for the modified glassy carbon plots in a 0.2 mM Fe^2+/3+^ redox system at 0.3 V (inset: Randles equivalent circuit).

The developed probes were characterized by electrochemical impedance spectroscopy. [Fe(CN)_6_]^3−/4−^ solution was chosen as the medium due to its well-known ease redox behavior. The potential at which to perform EIS studies was determined by cyclic voltammetry to be 0.3 V. The EIS parameters were obtained by fitting the Randles equivalent circuit (inset in Figure 4) to the experimental data and performing complex non-linear least-squares procedures available in an EIS data fitting computer program. The behavior of probes under electrochemical impedance studies is known to exhibit two sections: the circular part which is associated with resistance to charge transfer behavior and the straight line component associated with diffusion of redox materials toward the electrode surface (Wu et al., 2015). The higher the radius of the circle, the higher the difficulty with which the probe can facilitate redox reactions. The designed electrodes displayed different abilities to act as electron exchange platforms. The R_**CT**_ values in the above media varied in the following order: GONS/GCE (2.611 kΩ) > poly-CoTCPc-CoTAPc/GCE (2.207 kΩ) > CoTCPc/GCE (2.084 kΩ) > CoTAPc/GCE (1.901 kΩ) > CoTAPc-GONS/GCE (1.616 kΩ > GCE (1.179 kΩ) > poly-CoTCPc-CoTAPc-GONS/GCE (1.061 kΩ) (Figure 4). Similar results were obtained when the same investigation was done by cyclic voltammetry under the same conditions (figure not shown). The Bode plots also confirmed the modification of the glassy carbon electrodes, with phase angle shifting from 75° for GCE to 55° for poly-CoTCPc-CoTAPc-GONS/GCE and corresponding frequency decrease (Supplementary Figure S2), confirming efficient electron transfer and electrocatalysis after modification (Teo et al., 2021). It can be noted that the combination of the two differently substituted phthalocyanines in the absence of the electron-rich GONS compromised the electron transfer ability of the resultant electrodes, comparing poly-CoTCPc-CoTAPc/GCE (2.207 kΩ) with CoTCPc/GCE (2.084 kΩ) or CoTAPc/GCE (1.901 kΩ). The introduction of the graphene oxide moiety resulted in an improved electron transfer ability, comparing CoTAPc/GCE (1.901 kΩ) with CoTAPc-GONS/GCE (1.616 kΩ) and poly-CoTCPc-CoTAPc/GCE (2.207 kΩ) with poly-CoTCPc-CoTAPc-GONS/GCE (1.061 kΩ). This is attributable to the availability of abundant delocalized pi electron systems in sp^2^ carbon skeletons, for electrical conductivity (Nkhahle et al., 2019). Carboxy-terminated phthalocyanines have a poor electron transfer ability compared to amine-terminated counterparts. This shows that the pull (electron withdrawing) effect of the carboxyl group reduces the availability of the pi electron system on the central metal (redox center) for redox participation or otherwise for amine-terminated macrocycles as observed elsewhere (Nkhahle et al., 2019). To further explore the nature of the modified electrode surfaces, the effective electroactive surface was determined in the same media because of a favorable electron exchange ability and established as follows: GONS/GCE 0.076 cm^2^, poly-CoTCPc-CoTAPc/GCE 0.093 cm^2^, CoTCPc/GCE 0.096 cm^2^, CoTAPc/GCE 0.099 cm^2^, CoTAPc-GONS/GCE 0.116 cm^2^, and poly-CoTCPc-CoTAPc-GONS/GCE 0.134. These are improved surface areas from the geometrical area of a bare GCE of 0.071 cm^2^, showing a highly improved electroactive area for poly-CoTCPc-CoTAPc-GONS/GCE. For this reason, the latter is going to be studied in depth for the remainder of this work.

### Electrocatalytic Detection

Electrocatalytic detection (redox reactions) is usually a subject of a number of factors including pH, the amount of electrode modifier, and the method of modification among others. It is therefore apparent that these factors have to be interrogated thoroughly and optimized. The amount of modifier was optimized at 5 µL of 1 mg/ml of DMF using the drop dry method under inert conditions. The effect of pH was investigated in the range 4–8 as guided by previous investigations (Filik et al., 2016; Kumar Gupta et al., 2018). There was a significant variation of the background current and detection potentials in the detection of both ascorbic acid and tryptophan during the development of the sensor platform in this range of pH. Variations in pH values normally determine the extent of protonation or deprotonation of the analyte, hence its ease of oxidation or reduction which subsequently brings a signal of its presence or absence setting the basis of detection and quantification. Among other important parameters to consider, the kinetics, thermodynamics, and sensitivity of such reactions on the fabricated platform deserve much attention and set a good basis for the evaluation of the potential sensor. Robustness and stability of any sensor cannot be overlooked, and as such, this has to be addressed. A good detection platform has to be selective and free from common interferences for it to be of any practical relevance which is also a preoccupation of this work.

### pH Optimization

**FIGURE 5:**
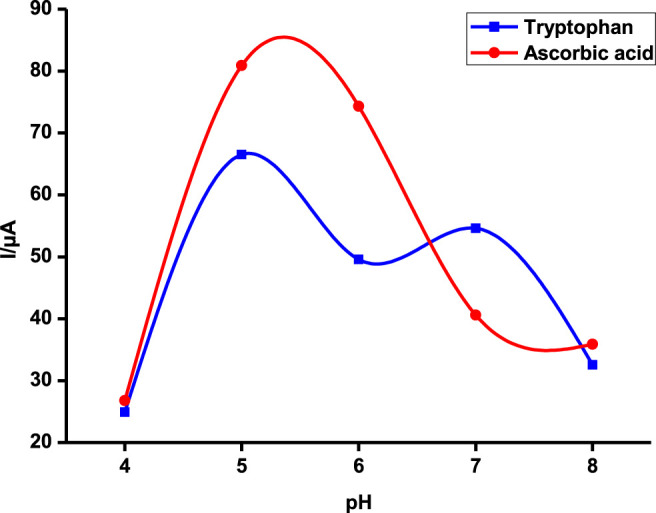
Detection current variation with changes in pH on poly-CoTCPc-CoTAPc-GONS/GCE in the buffer solution at a scan rate of 100 mV/s.

**SCHEME 2:**
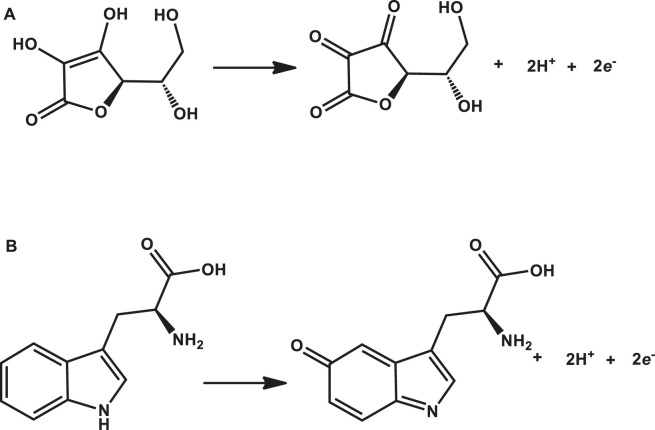
Mechanism of detection of ascorbic acid **(A)** and tryptophan **(B)** on poly-CoTCPc-CoTAPc-GONS/GCE, respectively.

Tryptophan and ascorbic acid were both detected at varying pHs, and the variation of both background detection currents and potentials was noted. The optimum detection currents were at pH 5 (Figure 5) for both tryptophan and ascorbic acid using a composite (poly-CoTCPc-CoTAPc-GONS/GCE) electrode due to its superior performance as established already in an efficient redox medium Fe^2+/3+^. Both analytes displayed a similar trend also observed elsewhere (Chihava et al., 2020) with other analytes: peak potentials decrease as the pH increases due to a proton transfer process. Scheme 2 shows the mechanism of detection of ascorbic acid and tryptophan on poly-CoTCPc-CoTAPc-GONS/GCE, respectively.

Both reactions display readiness to oxidation as observed on the poly-CoTCPc-CoTAPc-GONS/GCE platform. While the fabricated platforms are good candidates for the detection of the two residues, the poly-CoTCPc-CoTAPc-GONS/GCE platform has significantly improved background currents and energetically feasible detection potentials at pH 5 for both residues; hence, we report its performance here onward.

### Quantification of Tryptophan and Ascorbic Acid

**FIGURE 6:**
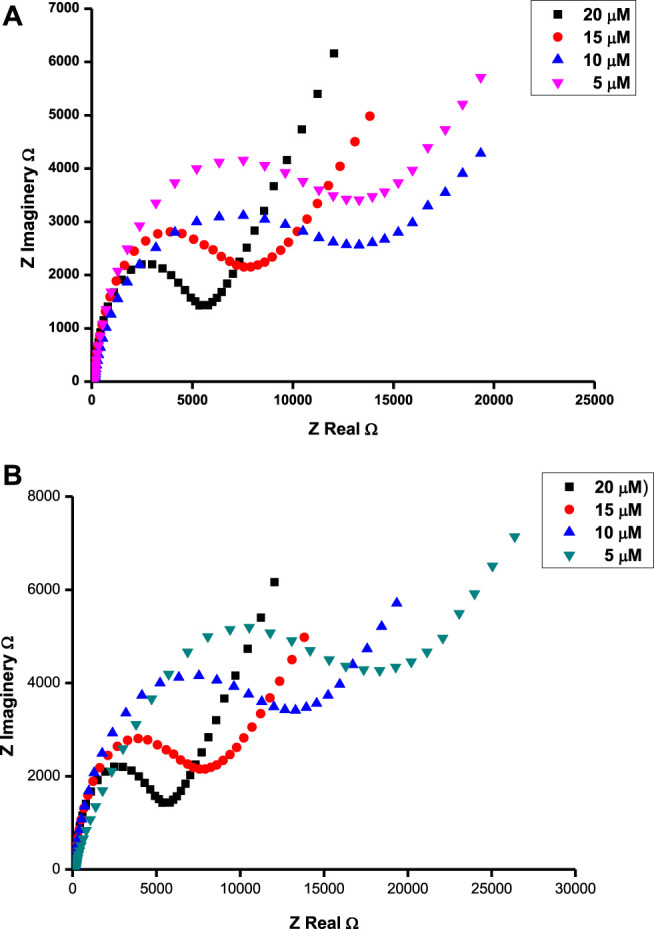
Nyquist plots for ascorbic acid at 0.37 V **(A)** and tryptophan at 0.72 V **(B)** in different concentrations of respective analytes on the surface of poly-CoTCPc-CoTAPc-GONS/GCE at pH 5.

**FIGURE 7:**
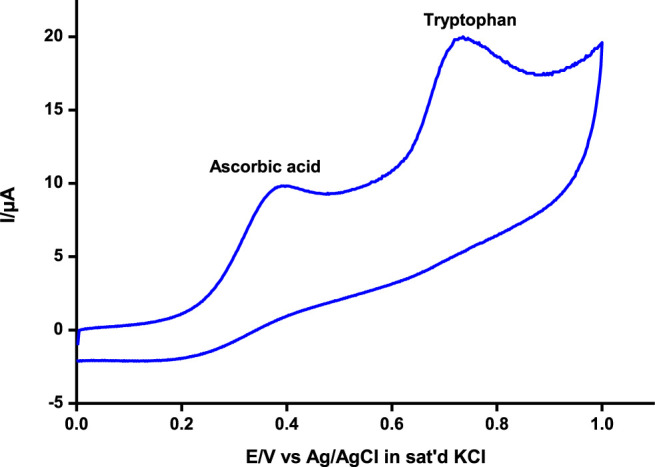
Simultaneous detection of ascorbic acid and tryptophan by cyclic voltammetry on poly-CoTCPc-CoTAPc-GONS/GCE at pH 5. Scan rate, 100 mV/s.

Very little work has been done in which EIS has been employed as a quantifying tool for the analyte. Electrochemical impedance spectroscopy utilizes the conductivity response of an electrolyte to the concentration of the conducting species, hence its beauty in analyte quantification. The assumption is that, within a certain range of concentrations, the conductivity should be proportional to the number of conducting species (Yang et al., 2014). The potential at which EIS studies were carried out was determined by cyclic voltammetry (Figure 7) as the corresponding oxidation peak. The EIS parameters were obtained by fitting the Randles equivalent circuit (inset in Figure 4) to the experimental data and performing complex non-linear least-squares procedures available in an EIS data fitting computer program. The resistance to charge transfer (R_CT_) for tryptophan on different sensor platforms varied in the following order: GONS/GCE (4.657 kΩ) > GCE (3.712 kΩ) > CoTAPc-GONS/GCE (2.842 kΩ) >CoTCPc/GCE (2.695 kΩ) > CoTAPc-GONS/GCE (2.561 kΩ) > poly-CoTCPc-CoTAPc/GCE (2.191 kΩ) > poly-CoTAPc-CoTAPc-GONS/GCE (1.254 kΩ) (Figure 6). A similar trend was also observed with the Bode plots and was characterized by varying phase angles and frequencies indicative of varying levels of effectiveness of electrode modification as shown in Supplementary Figure S3, for ascorbic acid as an example. The same order was also observed for the detection of ascorbic acid confirming the redox behavior established in Fe^2+/3+^ media; hence, poly-CoTAPc-CoTAPc-GONS/GCE will be utilized for further studies. Since this electrode showed very low resistance to charge, it was then used to study the response to concentration variation. Investigations showed that the resistance to charge linearly decreased with an increase in the concentration of the analyte in the range 0–20 µM of the analyte. This is evidence that the analyte is the source of current as a result of its oxidation.

Differential pulse and cyclic voltammetry were also employed to detect and quantify tryptophan at around 0.72 V and ascorbic acid at 0.37 V, respectively, similar to values reported elsewhere for independent and/or simultaneous detection of the analytes (Filik et al., 2016; Kumar Gupta et al., 2018; Govindasamy et al., 2019). The two analytes could be adequately detected simultaneously at significantly different potentials by both techniques as illustrated using cyclic voltammetry (Figure 7). Tryptophan and ascorbic acid are common residues in human physiological systems, hence the beauty of a platform such as poly-CoTAPc/CoTCPc/GONS/GCE reported in this work with the ability to detect the residues at significantly different potentials.

**FIGURE 8:**
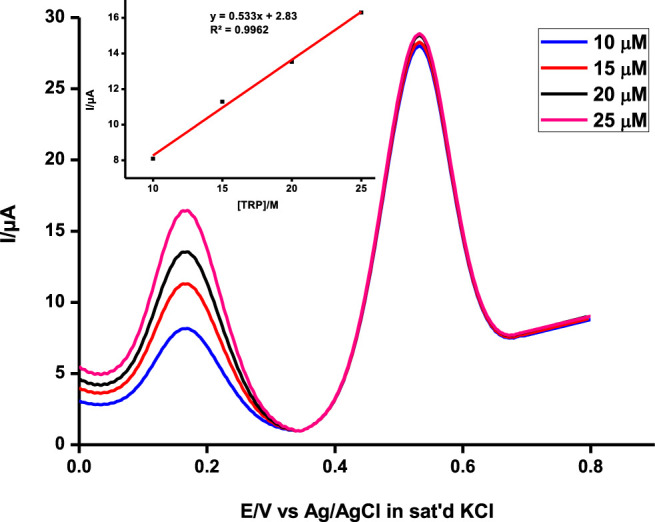
Simultaneous detection of a range of concentrations of ascorbic acid at a constant tryptophan concentration at pH 5 on poly-CoTCPc-CoTAPc-GONS/GCE. Scan rate, 100 mV/s.

This makes the probe very applicable in biological media since the two analytes coexist in physiological environments (Wu et al., 2015). To further ascertain non-interference between the two, the concentrations of the two were independently varied and the results were observed (Figure 8). The concentration of one analyte was increased, while the other was kept constant and vice versa, and the probe picked the difference with impressive sensitivity.

The developed sensing platform can therefore be used for simultaneous detection and quantification of the two analytes with reasonable reliability. It is also very important to establish the limits of detection and quantification of a sensing platform as biological molecules usually occur in minute concentrations. This was done using the 3δ and 10δ notations. The limit of detection and limit of quantification were found to be 2.1 × 10^−6^ and 5.5 × 10^−6^ M, respectively, for ascorbic acid and 1.65 × 10^−6^ and 5.5 × 10^−6^ M for tryptophan when the two analytes coexist comparable to reported values (Filik et al., 2016; Yao et al., 2020). These values have practical significance since they closely relate to physiological concentrations which lie in the range 28–99 mM for ascorbic acid (Hagel et al., 2018), and up to 95% of tryptophan is taken up by the kynurenine pathway (Kałużna-Czaplińska et al., 2019). Table 1 shows a comparison of poly-CoTCPc-CoTAPc-GONS/GCE with other reported electrodes (Ba et al., 2013; Yang et al., 2015; Filik et al., 2016; Wang et al., 2017) for simultaneous detection and shows its potential in application to real samples.

**TABLE 1:** Comparison of performance of poly-CoTCPc-CoTAPc-GONS/GCE with other electrodes reported in the literature.

Electrode	Analyte	Detection limit (µM)	Linear range (µM)	References
AzA/MWCNTs/AuNPs	Tryptophan	0.3	1–100	Filik et al. (2016)
NiCoO_2_/C/GCE	Tryptophan	5.7	0–943.4	Yang et al. (2015)
PSA/GCE	Tryptophan	0.0068	0.05–10	Ba et al. (2013)
CVD graphene	Tryptophan	0.1	0.25–75	Wang et al. (2017)
Poly-CoTCPc-CoTAPc-GONS/GCE	Tryptophan	1.65	5.5–80	This work
AzA/MWCNTs/AuNPs	Ascorbic acid	16	300–1000	Filik et al. (2016)
NiCoO_2_/C/GCE	Ascorbic acid	3.3	0–943.4	Yang et al. (2015)
CVD graphene	Ascorbic acid	1.58	5–1500	Wang et al. (2017)
Poly-CoTCPc-CoTAPc-GONS/GCE	Ascorbic acid	2.1	7.12–100	This work

### Kinetics

**FIGURE 9:**
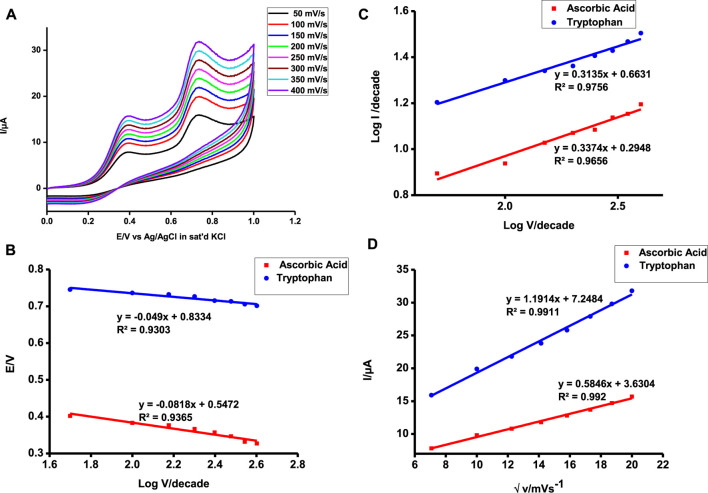
Scan rate of poly-CoTCPc-CoTAPc-GONS/GCE in 1 mM ascorbic acid and tryptophan from 50 to 400 mV **(A)**, V vs. log v **(B)**, log I vs. log E **(C)**, and plot of Ipa vs. √v **(D)**.

Since the probe under investigation could satisfactorily detect and quantify both analytes in a single solution, the behavior of detection was investigated at different scan rates simultaneously. For both analytes, it was observed that the peak position shifted with the change of scan rates indicative of an irreversible reaction (Sajjan et al., 2019). Irreversible reactions are governed by the following equation:

$$E_{p}=\frac{b}{2}\log v+k,$$ (1)

where *b* is the Tafel slope, v is the scan rate, and k is a constant. Tafel slopes of 162 and 98 mV/decade were obtained for ascorbic acid and tryptophan, respectively. Tafel slopes above 120 mV/decade imply slower reactions (Liu et al., 2020; Mooni et al., 2020), while those below are suggestive of diffusion-controlled reactions. Log v against log I were plotted and characterized by straight lines of curves of slopes below 0.5 further suggesting diffusion-controlled reactions for both ascorbic acid and tryptophan oxidation (Figure 9). Adsorption-characterized reactions have slopes close to unity, while gradients below 0.5 are characteristic of diffusion-controlled reactions (Shumba and Nyokong, 2016b). Diffusion-controlled reactions are facile by nature and hence characterized by very high slopes of the plots of peak current versus the square root of scan rate. Figures 9A–C shows such plots, and it is apparent that the developed probe is more facile during the oxidation of tryptophan than it is for ascorbic acid [slope 1.19 µA/(mVs^−1^)^1/2^ against 0.58 µA/(mVs^−1^)^1/2^] despite it having a higher activation energy as depicted by its detection potential.

The plots of Ipa vs. √v gave a linear plot evidence of diffusion-controlled reactions. Diffusion-controlled reactions are facile in nature indicative of a highly catalyzed reaction. The slope of the plot is indicative of how facile the reaction is. Tryptophan detection is more facile and readily feasible as shown by a higher slope in Figure 9D.

The Langmuir adsorption theory was applied to confirm the feasibility of the two oxidative reactions as reported earlier (Shumba and Nyokong, 2016c). The Gibbs free energy obtained was −17.45 and −14.83 kJ mol^−1^ for ascorbic acid and tryptophan, respectively, depicting a spontaneous reaction on the electrode surface. These values are comparable to those reported elsewhere for high Tafel slopes (Adekunle et al., 2011; Shumba and Nyokong, 2016c).

### Catalytic Rate Constants

**FIGURE 10:**
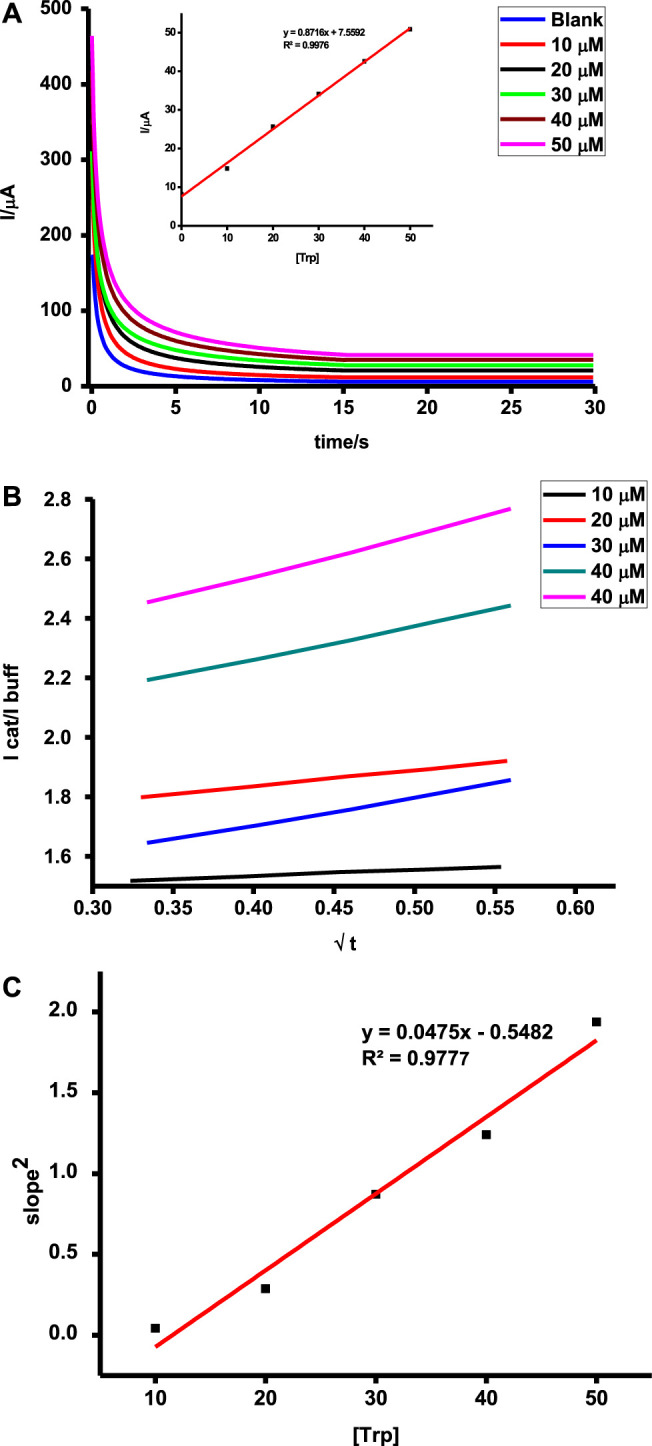
**(A)** Chronoamperometric scans (inset **=** dependence of tryptophan concentration on current); **(B)** plot of I_cat_/I_buf_ vs. t^1/2^; **(C)** plot of concentration of tryptophan vs. square of slopes on **(B)** determined using chronoamperometry for the poly-CoTAPc-CoTAPc-GO/GCE electrode.

Catalyzed reactions are characterized by improved reaction rates and low energy consumption reactions. The initial stages during chronoamperometric scans are characterized by rapid current decays before a constant signal is observed. This can be attributed to the oxidation of a high concentration of tryptophan or ascorbic acid as a result of adsorption followed by a steady diffusion-controlled step which reflects the actual concentration of the test analyte, hence higher currents for higher concentrations as shown in Figure 10. Oxidation of tryptophan and ascorbic acid on poly-CoTAPc-CoTCPc-GONS/GCE showed different sensitivities of 0.9 and 0.1 AM^−1^, respectively. A similar trend was also observed under DPV with the analyte detection giving sensitivities of 0.6 and 0.2 AM^−1^, respectively. This shows that the developed sensing platform is more responsive to tryptophan compared to ascorbic acid though the latter is detected at a more favorable potential.

Rate constants can be evaluated using

$$\frac{I_{cat}}{I_{buf}}=\frac{\gamma^{\frac{1}{2}}\left[\pi^{\frac{1}{2}}erf\left(\gamma^{\frac{1}{2}}\right)+\exp\left(-\gamma \right)\right]}{\gamma^{\frac{1}{2}}},$$ (2)

where $I_{cat}$ and $I_{buf}$ are the currents on poly-CoTAPc-CoTCPc-GONS/GCE in the presence and absence of the test analyte, respectively, and erf is the error function. At values of γ above 2, the error function is approximately equal to 1, and Eq. 2 reduces to

$$\frac{I_{cat}}{I_{buf}}=\gamma^{\frac{1}{2}}\pi^{\frac{1}{2}}=\pi^{\frac{1}{2}}{\left(kC_{o}t\right)}^{\frac{1}{2}},$$ (3)

where *k* is the catalytic rate constant, t is the time elapsed, and $\gamma =kC_{o}t$ ( $C_{o}$ is the bulk concentration of the test analyte). The plot of $\frac{I_{cat}}{I_{buf}}$ against $t^{\frac{1}{2}}$ therefore gives a slope of πk , and the catalytic rate constant can therefore be deduced from the plot of square of the above plot against concentration. The equations of the plots are represented as

$$y=0.0475\left[Trp\right]\frac{s^{-1}}{\mu M}+0.582,$$ (4)

$$y=0.0585\left[AA\right]\frac{s^{-1}}{\mu M}-1.634.$$ (5)

These translate to catalytic rate constants of 1.512 × 10^4^ and 1.862 × 10^4^ M^−1^s^−1^ for tryptophan and ascorbic acid on the modified electrode, respectively. Such values are attractive and pose the probe as a potential candidate for practical applications.

### Practical Application of the Probes

**FIGURE 11:**
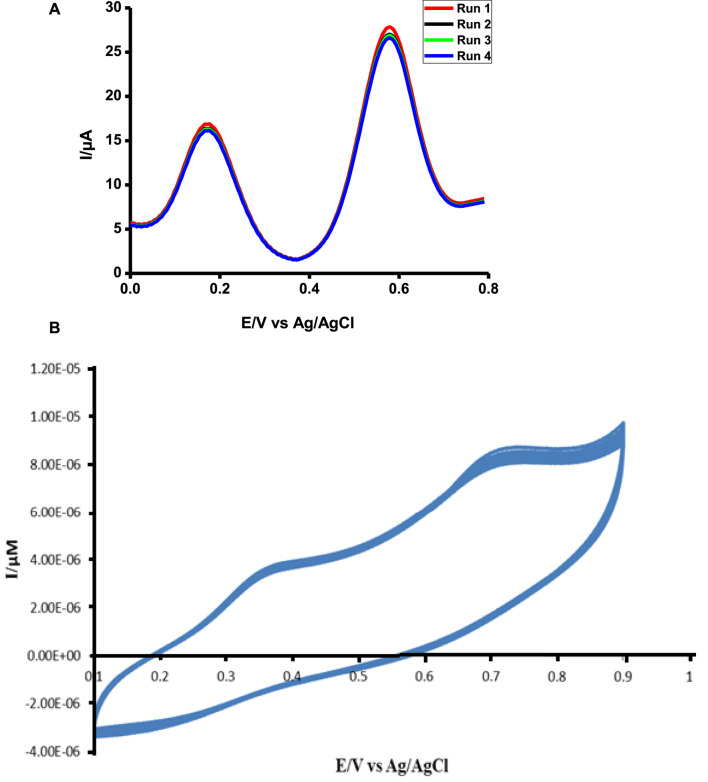
Stability study of poly-CoTCPc-CoTAPc-GONS/GCE in 1 mM ascorbic acid and tryptophan in pH 5 buffer solution using DPV **(A)** and CV **(B)**.

Most probes usually work well in synthetic solutions but simply get poisoned or lose selectivity once in real samples. The prepared probe was tested for selectivity in the presence of other interferences and in milk samples. The standard addition method was used to investigate the recoveries of ascorbic acid in milk samples and tryptophan in urine samples, Table 2. The recoveries were quite impressive: 95–106.7% for ascorbic acid and 91.7–105% for tryptophan comparable to and even better than the values reported elsewhere (Govindasamy et al., 2019), implying that the developed probe is a promising candidate for the sensing of these biomolecules. With very high accuracy above 97%, low relative standard deviation below 6%, and standard error in the nanorange (Table 3), the probe provides a high potential as a sensor platform for both tryptophan and ascorbic acid in real samples. The probes show no interferences of potentially electroactive molecules in milk samples, as shown in Figure 11.

**TABLE 2:** Determination of ascorbic acid and tryptophan in milk and urine samples using poly-CoTCPc-CoTAPc-GONS/GCE.

Sample	Electrode	Analyte	Original (µM)	Spiked (µM)	Found (µM)	Recovery (%)
Milk	CoTAPc-CoTCPc-GONS-GCE	Ascorbic acid	1.5	10	11.6	106.7
			3	20	22.9	96.7
*Urine	Nafion/AuNPs/AzA/MWCNTs-GCE	Tryptophan	6	30	35.7	95.0
			—	500	498.6	99.72
			2	10	12.1	105.0
			4	20	23.9	97.5
*Urine	Nafion/AuNPs/AzA/MWCNTs-GCE		6	30	35.5	91.7
**Aminoven 10%	Nafion/AuNPs/AzA/MWCNTs-GCE		190	50	240	100
	GNP/CILE		—	10	10.1	101
			100	40	143.9	100.6

*Filik et al. (2016); **Liu et al. (2020).

**TABLE 3:** Validation parameters.

Analytical method	RSD	SE of slope	SE of intercept	Accuracy
Chronoamperometry	6.56	2.86E-08	9.48E-07	97.37 ± 6.38
Tryptophan Ascorbic acid	3.67	3.26E-09	1.08E-07	116.98 ± 4.30
DPV at constant	3.12	2.50E-08	4.59E-07	99.85 ± 3.12
Tryptophan Ascorbic acid	1.55	5.24E-08	9.63E-07	99.60 ± 1.55

The probe was very stable after repetitive scans for both analytes (Figure 11). This is a very good sign showing that the probe can be used several times without signal loss; hence, it is a perfect candidate for practical use. While DPV shows stability over a period of 6 months when kept at 4°C and dry conditions, CV shows stability against repetitive scans.

## Conclusion

Polymerization of phthalocyanine entities has resulted in electron redistribution patterns within the modifiers due to the different electron withdrawal affinities resulting from the new bonding patterns. This has significantly improved the electrocatalytic behavior of the designed sensor posing a very promising multi-analyte platform for the detection of coexisting pollutants. The need for minimal to no sample preparation is a very positive aspect in the field of sensor development. Coupled with low detection limits, resistance to fouling, robustness, and high selectivity, the developed sensor has high potential as a practical solution to environmental pollution monitoring.

## Data Availability

The raw data supporting the conclusion of this article will be made available by the authors, without undue reservation.
