# Supplementary material for: Poly-Phthalocyanine–Doped Graphene Oxide Nanosheet Conjugates for Electrocatalytic Oxidation of Drug Residues
Source: Front Chem. 2021 Dec 7;9:633547. doi: 10.3389/fchem.2021.633547 (PMC8688842; doi:10.3389/fchem.2021.633547)
Supplement: Supplementary file 1 [file DataSheet1.pdf]

Supplementary Information

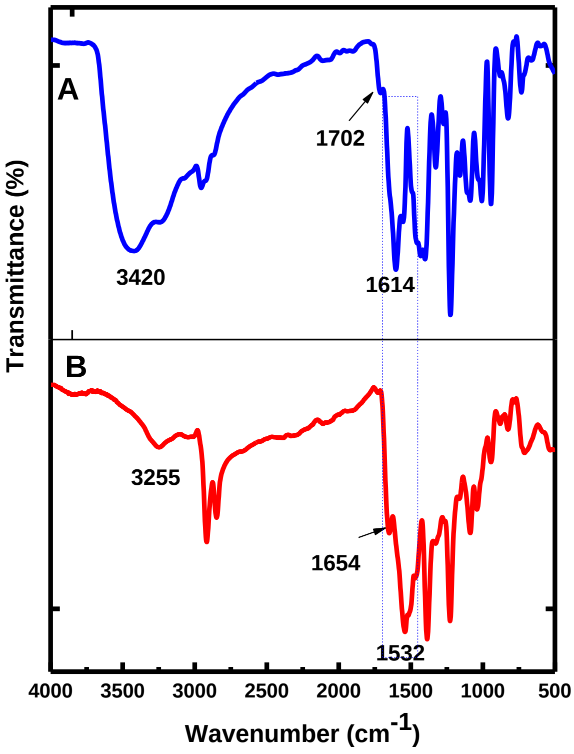

Fig. S1 FT-IR spectra of CoTCPc (A) and poly-CoTCPc-CoTAPc (B).

Bode plots

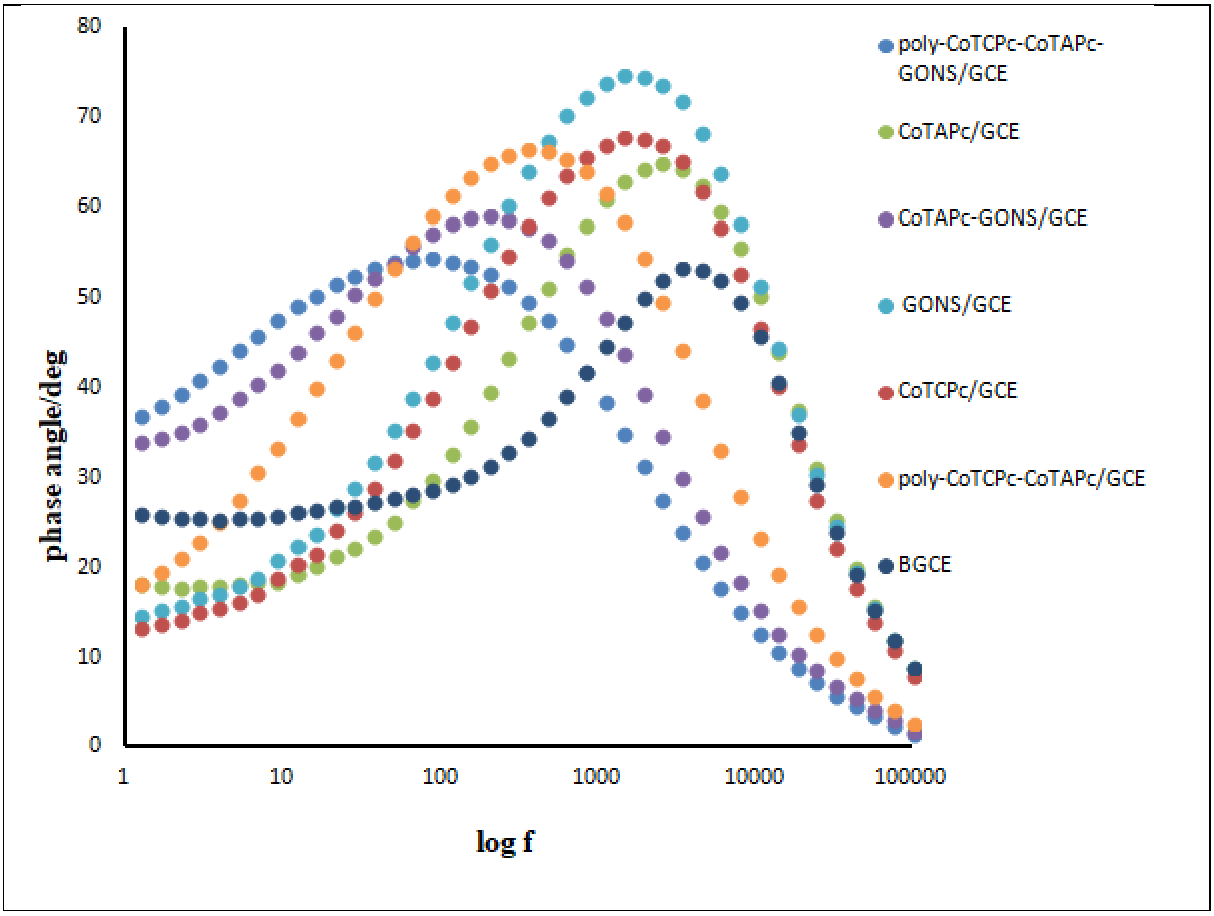

Fig. S2. Bode plots in 1 mM [Fe $(CN)_6$ $]^{-3/-4}$ in 0.1 M KCl solution for GCE, GONS/GCE, CoTCPc/GCE, CoTAPc/GCE, CoTAPc-GONS/GCE, poly-CoTCPc-CoTAPc/GCE and poly-CoTCPc-CoTAPc-GONS/GCE.

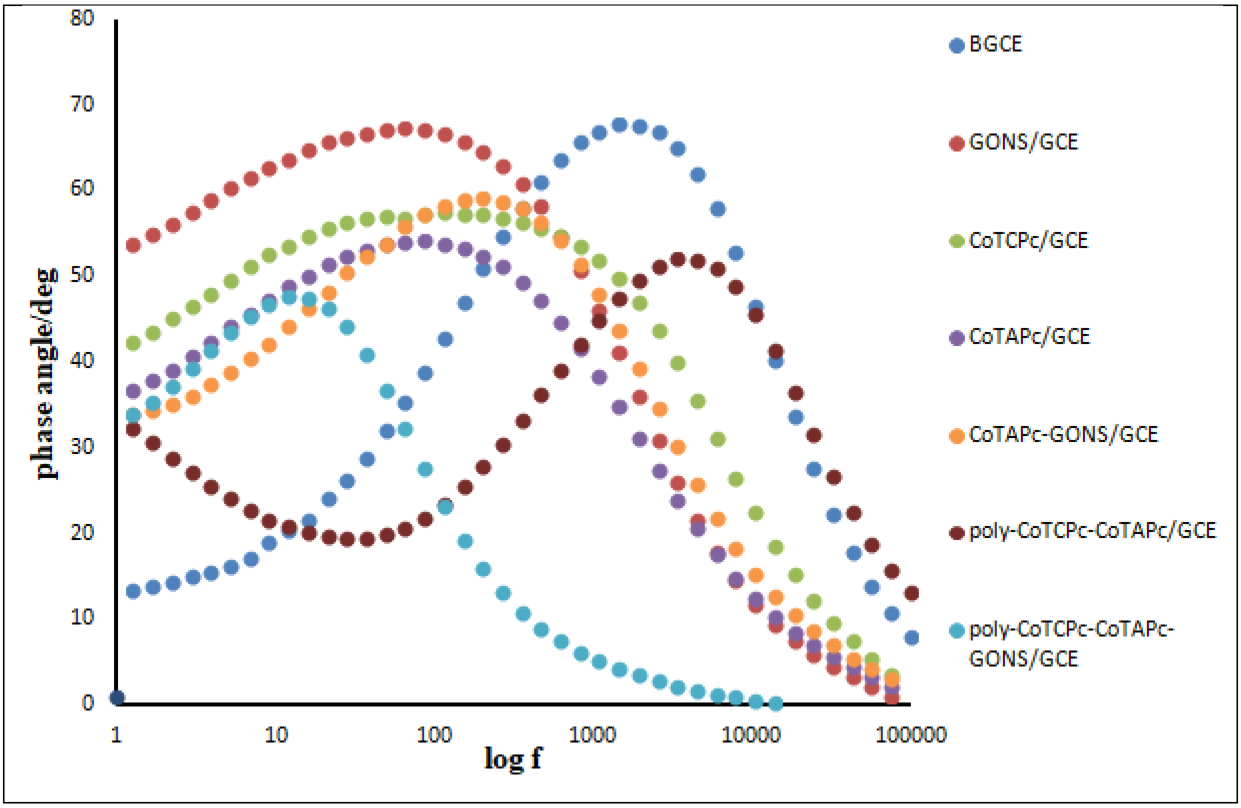

Fig. S3 Bode plots for different electrodes in 1 mM ascorbic acid.

In the presence of tryptophan, poly-CoTCPc-CoTAPc-GO/GCE has better electrocatalytic properties as compared to the other electrodes. This is shown by the poly-CoTCPc-CoTAPc-GO/GCE lower phase angle of 54.36 ° at a lower frequency as compared to the other electrodes, **Fig 1c** with phase angles of GCE 74.60 °, GONS/GCE 71.92 °, CoTAPc-GONS/GCE 66.35 °, CoTCPc-CoTAPc/GCE 62.73 °, CoTCPc/GCE 62.69 ° and CoTAPc/GCE 57.36 °.

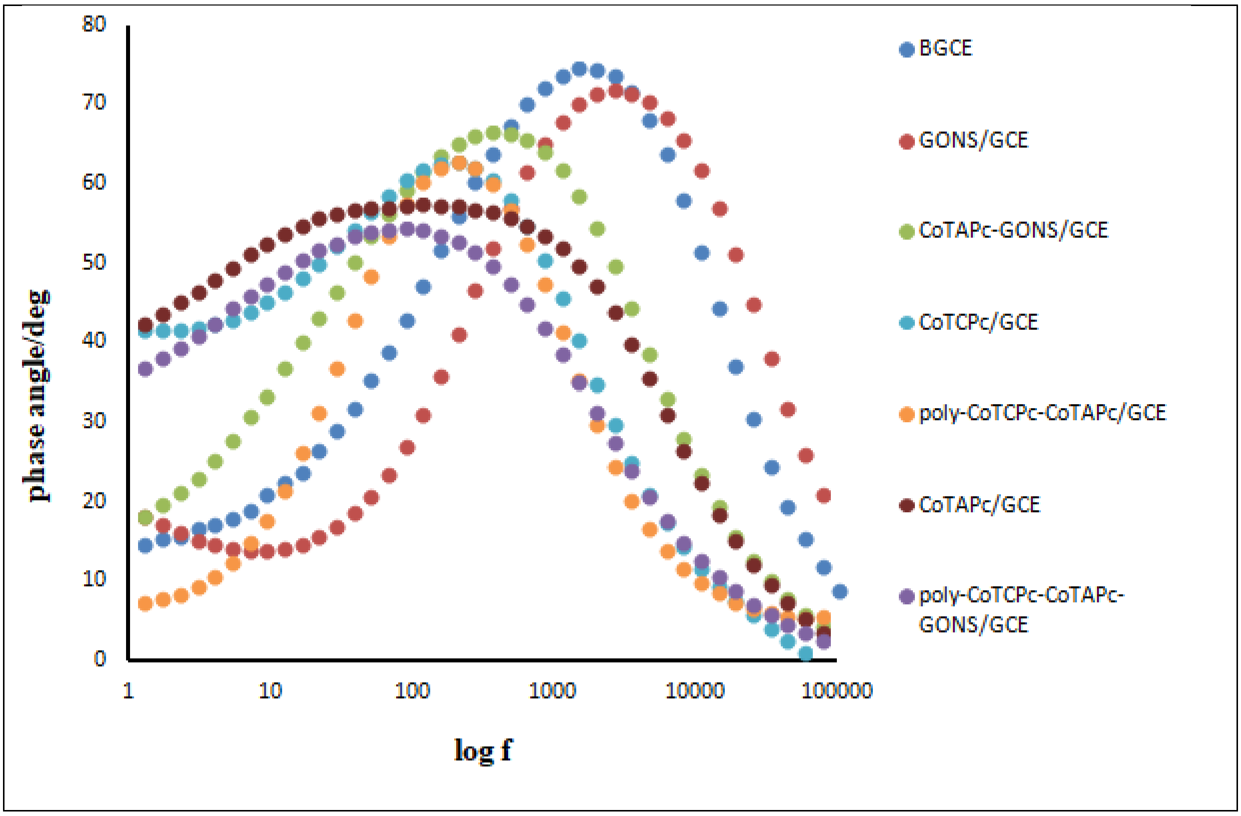

Fig. S4 Bode plots for different electrodes in 1mM tryptophan.
